# Cinchonidine induces muscle weakness by inhibiting insulin-mediated IRS-1-AKT signaling pathway

**DOI:** 10.71150/jm.2511017

**Published:** 2025-12-31

**Authors:** Mi Ran Byun, Sang Hoon Joo, Young-Suk Jung, Joon-Seok Choi

**Affiliations:** 1 Department of Pharmacy, Daegu Catholic University, Gyeongsan 38430, Republic of Korea; 2 Department of Pharmacy, College of Pharmacy, Research Institute for Drug Development, Pusan National University, Busan 46241, Republic of Korea

**Keywords:** sarcopenia, muscle regeneration, muscle differentiation, cinchonidine, IRS-1

## Abstract

Sarcopenia is an age-related condition marked by a reduction in muscle mass and strength, and it is associated with impaired muscle regeneration and differentiation. While diseases like cardiovascular and chronic liver disease can induce sarcopenia, there is limited evidence regarding the specific diseases and mechanisms responsible for its development. In skeletal muscle, the loss of muscle mass is accompanied by a decrease in myofilament proteins and the inhibition of muscle differentiation in satellite cells. Bioactive compounds obtained from natural products have been traditionally used as therapeutics for diverse conditions. In this report, we investigated the effect of cinchonidine (CD) extracted from Cinchona tree on muscle differentiation of mouse satellite cells, and myoblast cell lines. CD significantly inhibited muscle differentiation by suppressing myotube formation and gene expression of myogenesis markers. In addition, CD reduced muscle differentiation by blocking phosphorylation of insulin receptor substrate 1 (IRS-1) during insulin-induced signal transduction. Therefore, the results show that CD, an antimalarial agent, inhibited muscle differentiation through the suppression of IRS-1 phosphorylation, suggesting that sarcopenia can be induced by CD.

## Introduction

Muscle weakness refers to a decrease in muscle mass and strength that is usually related with aging. As aging progresses, the skeletal muscle mass of the human body decreases by about 0.1 to 0.5% annually from the age of 30, and muscle loss accelerates after the age of 60 (Curcio et al., 2016; Volpi et al., 2004). Muscle loss due to aging is called 'sarcopenia' (Mithal et al., 2013; Rosenberg, 1997), which closely correlates with various conditions including osteoporosis (Di Monaco et al., 2022; Pan and Xu, 2022), fractures (Yeung et al., 2019), diabetes (Mesinovic and Scott, 2019; Mesinovic et al., 2019), and cardiovascular disease (Bielecka-Dabrowa et al., 2020). Sarcopenia can be attributed to various factors, including aging, insufficient exercise, diseases, and inflammation (Huo et al., 2022; Karim et al., 2024; Walston, 2012). Sarcopenia can also be induced by medications such as antineoplastic agents and hypoglycemic agents. The sarcopenia resulting from these treatments is reported to show as a secondary effect due to alterations in muscle protein metabolism, protein synthesis, and protein degradation induced by the drugs (Bozzetti, 2020; Kuzuya, 2024; Massimino et al., 2021; Pedrosa et al., 2023). However, it has not been confirmed whether these medications affect muscle differentiation.

The process of myogenesis commences with the proliferation of mononuclear myoblasts and the expression of genes that trigger muscle differentiation, such as myosin heavy chain (MHC), MyoD, and myogenin, etc. (Bentzinger et al., 2012; Buckingham, 2006). Subsequently, the cells fuse with each other to form the multinucleated myotubes and muscle fibers (Abmayr and Pavlath, 2012). In matured adult muscle tissue, the process of muscle regeneration begins with muscle stem cells, known as muscle satellite cells. These satellite cells normally remain in a quiescent state. However, when exposed to a muscle-damaged environment, they break out of their quiescent state and become activated. Then, the cells amplify and differentiate into myoblasts, ultimately contributing to the formation of new muscle fibers (Birbrair and Delbono, 2015; Kadi et al., 2004). Myogenic differentiation is regulated by several pathways (Relaix et al., 2021). In particular, insulin signaling plays an essential role in myogenic differentiation. When insulin binds to its receptor, it activates the downstream signaling factor insulin receptor substrate (IRS). IRS subsequently triggers different signaling pathways including MAPK (Van Gerrewey and Chung, 2024), AKT/PKB, and mTOR pathways to promote muscle regeneration and differentiation. The meticulous signaling by IRS is of paramount importance in ensuring the accurate relay of insulin-induced AKT-mTOR activation (Schiaffino and Mammucari, 2011; Sun et al., 1991).

Cinchonidine (CD) and its isomers Cinchonine (CN) are alkaloid compounds found in the bark of trees such as *Cinchona*, *Cinchona officinalis*, and *Cinchona pubescens*, and have been used as an anti-malarial (Warhurst, 1981). CN is known to mediate multi-drug resistance and improve cisplatin-induced ototoxicity (Ruiz-Mesia et al., 2005; Tang et al., 2023). In addition, anti-platelet (Shah et al., 1998) and anti-obesity (Jung et al., 2012) effects have been reported. Recent studies have indicated that CN induces osteogenic differentiation while also inhibiting osteoclastogenesis (Jo et al., 2021). This prompted us to investigate the effect of CD on mesenchymal stem cell differentiation, with a specific focus on the role of CD in myogenic differentiation. In this study, we challenged the mouse myoblast C2C12 cells with CD to see if CD inhibited myogenic differentiation of C2C12 cells and determined the mechanisms by which CD inhibits myogenic differentiation through inhibiting IRS-1-AKT signaling.

## Materials and Methods

### Cell culture and myogenic differentiation

C2C12 cells were maintained in Dulbecco’s Modified Eagle Medium (DMEM) containing 20% fetal bovine serum and 1% antibiotics (Cytiva, USA). To induce myogenic differentiation, C2C12 cells were plated at a density of 2 × 10^5^ cells in 6-well plates and incubated for 2 days. Subsequently, culture media were changed to differentiation media, i.e., DMEM containing 2% horse serum (Gibco, Thermo Fisher Scientific Inc., USA). The differentiation media were replaced every 2 days.

### Western blot analysis

Differentiated cells were lysed in RIPA lysis buffer (Biosesang, Korea) containing 50 mM Tris-HCl (pH 7.5), 150 mM NaCl, 1% Triton X-100, 0.1% SDS, 1% sodium deoxycholate, 2 mM EDTA and protease inhibitor cocktail (Sigma-Aldrich, USA). The cell lysates were denatured with SDS sample buffer, resolved by SDS-PAGE, transferred to 0.45 μm PVDF membrane. The protein-bound membranes were incubated with anti-MHC (Myosin heavy chain, Developmental Studies Hybridoma Bank, USA) and anti-β-actin (Sigma-Aldrich, USA) antibodies. For insulin signaling assay, C2C12 cells were seeded on 6-well plates at a density of 2 × 10^4^ cells/cm^2^. When the cells reached 80% confluency, the cells were washed with PBS and media was changed with DMEM containing 0.1% FBS and incubated for 16 h. The cells cultured in serum-deprived media were treated with 5 ng/ml insulin for 15 min and the protein level was analyzed using specific primary antibodies.

### Animals

To confirm the muscle regeneration effect of cinchonidine, 50 μl of 1.2% BaCl_2_ was injected into the left tibialis anterior (TA) muscle of 8-week-old C57BL/6 male mice, and the following day, mice with muscle injury were administered with 25 mg/kg of cinchonidine, which was diluted in drinking water, for 6 days. Subsequently, the mice were dissected, and hematoxylin and eosin (H&E) staining of TA muscle was performed to confirm muscle regeneration.

### Muscle stem cell isolation

Mouse satellite cells (MSC) were isolated from tibialis anterior (TA) muscle of 8-week-old C57BL/6 mice. For MSC isolation, 50 μl of 1.2% BaCl_2_ was injected into the mouse TA muscle, and after 3 days, the mice were dissected and TA muscle was isolated. Each TA muscle was placed in a 5 ml of 0.2% collagenase type 2 solution, and then incubated at 37℃ for 1 h. Following the incubation, the TA muscle were triturated using a 20-gauge needle to obtain single muscle fibers, and single muscle fibers were collected using a centrifuge. Then, the fibers were washed with HAM’S/F-10 Nutrient mixture media, and resuspended with HAM’S/F-10 Nutrient mixture media containing 20% horse serum, 100 units/ml penicillin, 100 mg/ml streptomycin, and 5 ng/ml recombinant human FGF-basic (Peprotech, Thermo Fisher Scientific Inc., USA). To induce myogenic differentiation, the cells were incubated in DMEM supplemented with 2% horse serum.

### Real time PCR analysis

Total RNA was isolated using TRIzol reagent (Thermo Fisher Scientific Inc., USA) and cDNA was synthesized with Maxima RT PreMix (Oligo (dT)15 Primer) Reverse transcriptase (iNtRON Biotechnology, Korea). mRNA expression level of MCK, myogenin, and GAPDH in differentiated cells was analyzed by quantitative RT-PCR (CFX Connect Real-Time PCR Detection System, Bio-Rad, USA). PCR primer sequences were as Table 1.

### Cell viability assay (WST-water-soluble tetrazolium assay)

To test cell growth and viability, C2C12 cells were plated (5 × 10^3^ cells/well) on 96-well culture plate, grown for 24 h, and treated with cinchonidine 0, 10, 20, 50, and 100 μM in differentiation media or growth media. After 48 h of cinchonidine treatment, QuantiMax^TM^ WST assay reagent (BioMax, Korea) were added to the cells, and absorbance was measured at 450 nm using a microplate reader (BMG Labtech, Germany) to assess cell growth and viability.

### Immunocytochemistry

For immunofluorescence analysis, a 12-mm cover glass was placed in each well of a 24-well culture plate, and 4 × 10^4^ cells were seeded in each well. After differentiation, the cells were washed with PBS, fixed with 4% paraformaldehyde for 15 min, permeabilized with 0.05% Triton X-100 for 10 min, blocked with 3% bovine serum albumin, and stained with MHC antibody at 4℃ overnight. Subsequently, the cells were washed three times with ice-cold PBS, incubated with Alexa Fluor^TM^ 488 anti-mouse IgG (Thermo Fisher Scientific Inc., USA) secondary antibodies at room temperature for 90 min, and counterstained with DAPI (Vector Laboratories, USA). The fluorescence signals were analyzed using confocal microscopy.

### Statistical analysis

*P* values are presented as the ± Standard Error of the Mean and data were statistically analyzed using Student's *t*-test. A *p*-value of less than 0.05 was considered statistically significant.

## Results

### Cinchonidine (CD) does not exert cytotoxicity toward myoblast C2C12 cells under proliferation or differentiation conditions

**Fig. 1.**
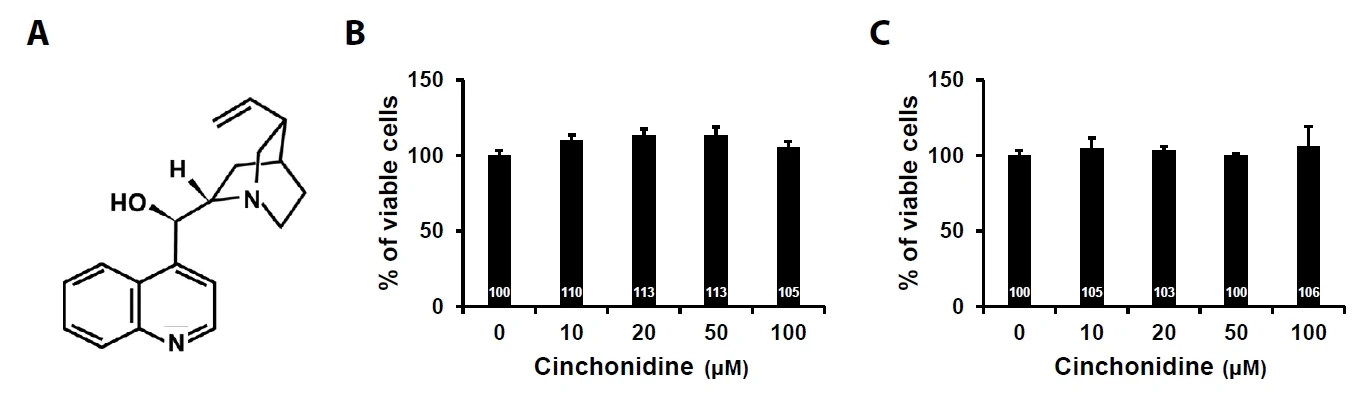
Cytotoxicity of CD under the cell growth and differentiation conditions. (A) Structure of Cinchonidine (CD). (B, C) Mouse myoblast cells were seeded in 96-well culture plates at a density of 5 × 10^3^ cells/well. After 24 h, the cells were cultured in either growth medium (B), or differentiated medium (C) with 0, 10, 20, 50, or 100 μM of CD for 48 h. Cell viability was assessed using the WST assay. The number represents the percentage of viable cells in the CD-treated group compared to the vehicle-treated group.

To determine the effect on the cell viability by cinchonidine (Fig. 1A), C2C12 cells were exposed to CD at concentrations of 0, 10, 20, 50, and 100 μM for 48 h, and the cell viability was measured using WST-1, a water-soluble tetrazolium salt. As shown in Fig. 1B, C2C12 cells treated with CD for 48 h showed cell viability as high as control cells. Similarly, C2C12 cells grown in the differentiation condition were not affected by the presence of CD (Fig. 1C). In both conditions, compared to the control, no cytotoxicity was observed in the CD-treated condition, and it was suggested that CD does not affect the cell viability of C2C12 cells. While not statistically significant, the viability of C2C12 cells treated with 100 μM CD seemed a little bit lower compared to the cells treated with lower concentrations, and we used 50 μM or lower concentrations of CD later on.

### CD inhibits myogenic differentiation of C2C12 cells

**Fig. 2.**
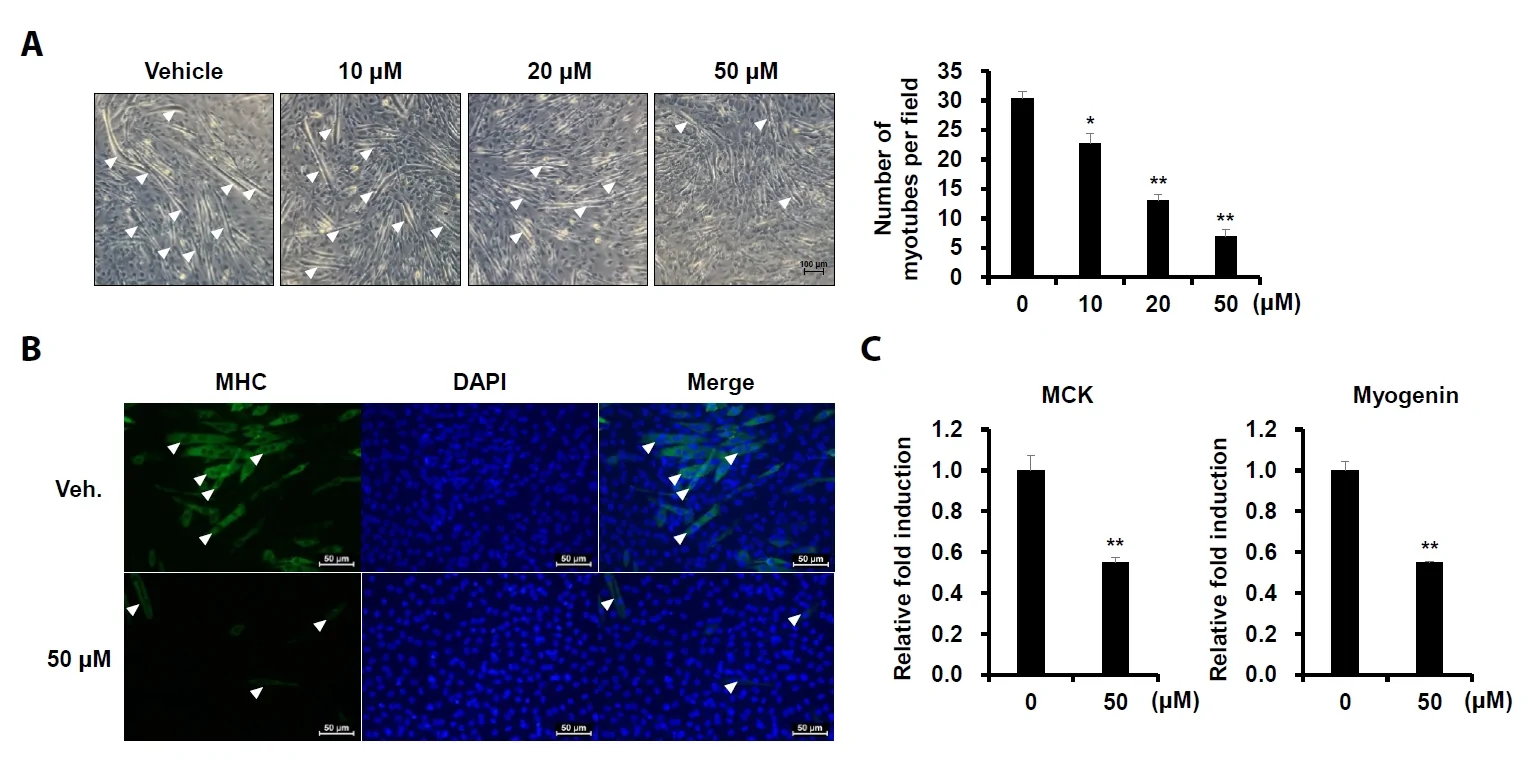
Myogenic differentiation. (A) C2C12 cells were differentiated into myotubes in the presence of indicated concentrations of CD for 2 days. The white arrows in the image indicate the formed myotubes. The right panel shows the quantification of myotubes in the left panel image. The number of myotubes in each-treated group was counted after observing myotubes in three different fields. ^*^P < 0.05, ^**^P < 0.001 *t*-test. (B) C2C12 cells were cultured in a differentiation medium containing vehicle or 50 μM of CD for 2 days. The differentiated cells were then subjected to immunocytochemistry analysis. Green fluorescent signals represent MHC expression, a marker for myogenic differentiation, while DAPI staining represents cell nuclei. The white arrows were used to indicate multinucleated cells. (C) Total RNA in (A) was harvested and reverse transcription and real-time PCR analysis were performed. Relative mRNA expression of MCK and myogenin were determined after normalization to mouse GAPDH expression. ^**^P < 0.001 *t*-test.

C2C12 cells, originally mononuclear and spindle-shaped myoblasts, may differentiate into myocytes to fuse and form multinucleated, elongated, and fibrous myotubes (Wong et al., 2020). To study the role of CD in myogenic differentiation, C2C12 cells undergoing differentiation were treated with CD. As the concentration of CD increased, the number of myotubes observed in the microscopic field decreased (Fig. 2A). To determine the MHC expression and formation of multinucleated cells after inducing myogenic differentiation, immunocytochemical analysis was also performed. In Fig. 2B, compared to control cells, CD-treated cells showed a significant decrease in MHC expression (green fluorescence). Additionally, myotubes with fused nuclei were observed forming at locations of the green fluorescence signal. In addition, the gene expression of muscle creatine kinase (MCK) and myogenin were decreased by CD treatment (Fig. 2C). Taken together, these results suggest that the CD inhibits myogenic differentiation of C2C12 cells.

### CD inactivates insulin signaling pathway in myogenic differentiation

**Fig. 3.**
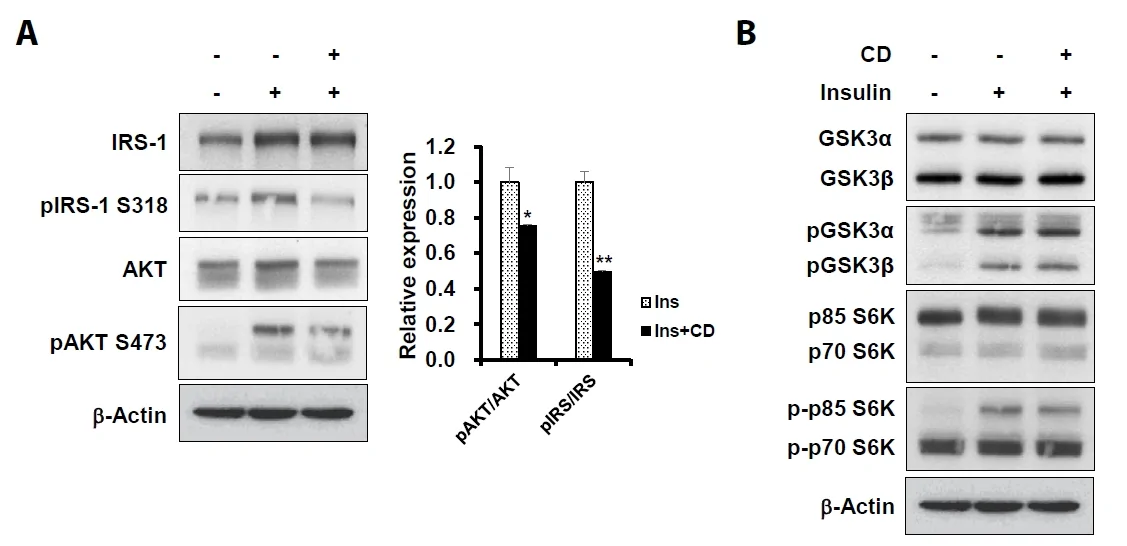
IRS-1/AKT signaling for myogenic differentiation. (A) C2C12 cells were incubated for 15 min with 5 ng/ml insulin in a serum-deprived medium containing vehicle or 50 μM of CD. The cells were subsequently lysed and analyzed for the levels of IRS-1, phospho-IRS-1, AKT, and phospho-AKT by immunoblot analysis. The graph on the right shows the relative expression of phosphorylated AKT and phosphorylated IRS, normalized to total AKT and total IRS, respectively. ^*^P < 0.05, ^**^P < 0.001 *t*-test. (B) Total cell lysates in (A) were analyzed using the indicated antibodies.

To study the mechanism by which CD regulates myogenic differentiation, C2C12 cells were treated with 50 μM of CD in the presence of insulin for 15 min, and the phosphorylation of IRS-1 and AKT protein was monitored. Interestingly, the phosphorylation of IRS-1, which was increased by insulin, decreased significantly with CD treatment, and the level of phosphorylated AKT also decreased (Fig. 3A). These results suggest that CD perturbs the IRS-1/AKT signaling pathway to inhibit the myogenic differentiation. In addition, we monitored the level of phosphorylation in p70/p85 S6 kinases (S6Ks) and α and β isoforms of glycogen synthase kinase 3 (GSK3). Unlike IRS-1 and AKT, the phosphorylation of S6Ks and GSK3 was not affected by the presence of CD (Fig. 3B).

### CD inhibits satellite cells activity and lowers muscle regeneration capacity

**Fig. 4.**
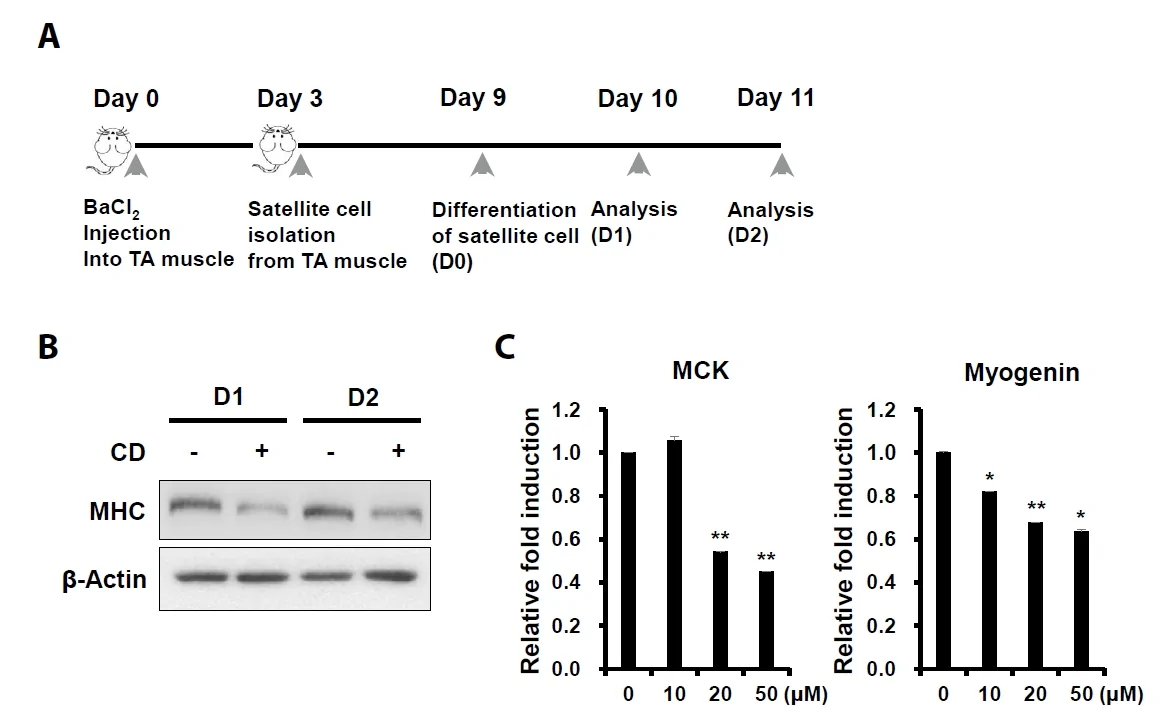
Myogenic differentiation activity of satellite cells. (A–C) BaCl_2_ was injected into the mouse TA muscle to induce muscle injury. 3 days later, muscle satellite cells were isolated from the TA muscle. Satellite cells were seeded in a 12-well culture plates and allowed to stabilize. The cells were subsequently differentiated into myotubes in the presence or absence of CD (50 μM) for 1 (D1) or 2 (D2) days. The degree of differentiation was compared by analyzing the expression level of MCK and myogenin using qRT-PCR. (A) Experimental design. (B) Western blot analysis of MHC expression in differentiated muscle satellite cells on D1 and D2. (C) Relative fold induction of MCK and myogenin in differentiated muscle satellite cells on D1 and D2. The relative expression of the indicated gene was normalized to GAPDH expression. ^*^P < 0.05, ^**^P < 0.001 *t*-test.

Muscle satellite cells exist in mature muscles and have the potential to regenerate muscle. Satellite cells are precursors that form skeletal muscle. When muscle cells are damaged, quiescent satellite cells get activated and differentiated into myocytes, myotubes and muscle fibers, thereby regenerating the muscle (Birbrair and Delbono, 2015; Kadi et al., 2004). To investigate the role of CD in the mouse myogenic differentiation model, satellite cells, isolated from tibial anterior muscles after BaCl_2_ injection, were allowed to differentiate in the presence of either CD or vehicle for 24 and 48 h (Fig. 4A). The expression of MHC was significantly decreased in the CD treatment group compared to the vehicle treatment group (Fig. 4B). In addition, the expression of MCH and myogenin decreased as the concentration of CD increased (Fig. 4C).

**Fig. 5.**
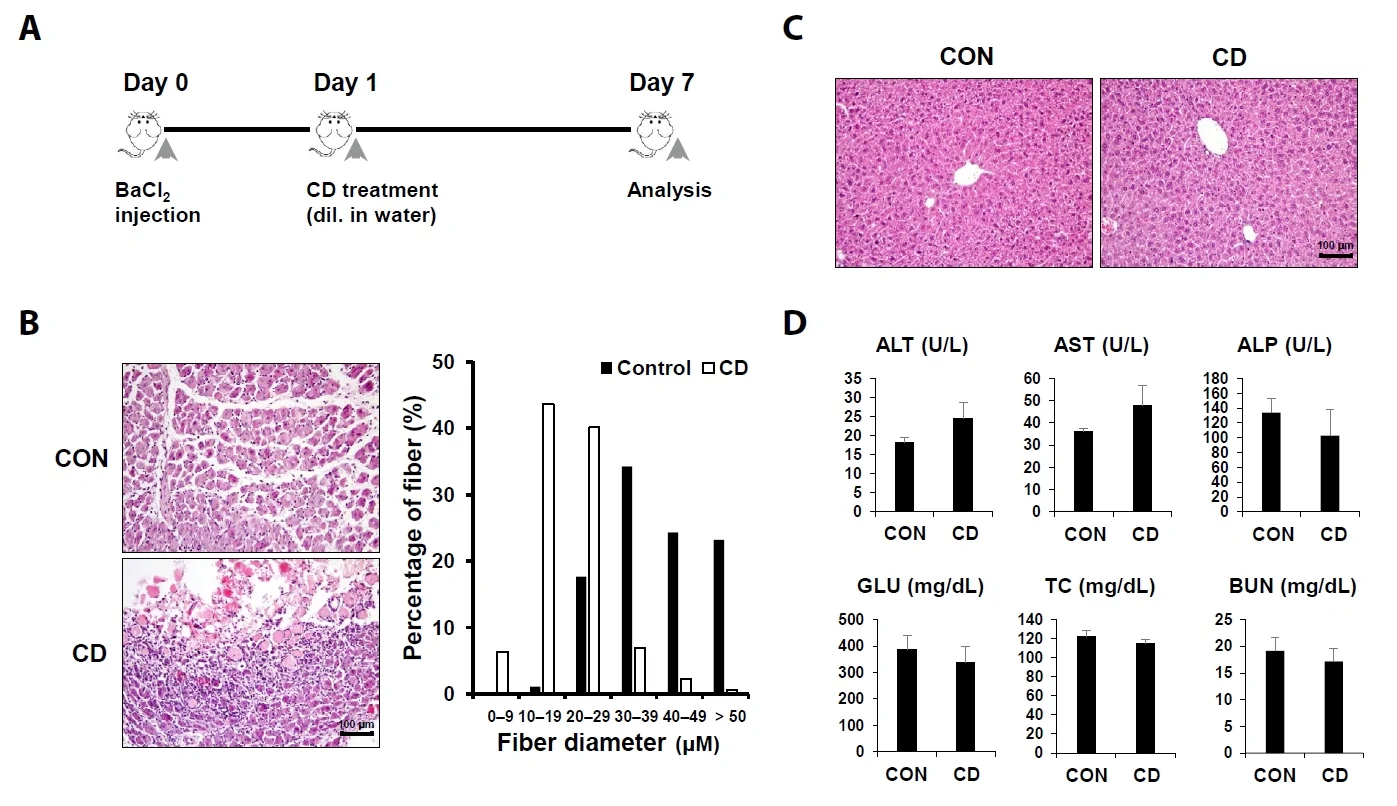
Muscle regeneration in a mouse model. (A) The experimental design of CD treatment for muscle regeneration in vivo. 50 μl of BaCl_2_ injected into mouse left TA muscle. And the following day, CD diluted in drinking water is administered. After six days, blood and liver were collected for toxicity assessment, and TA muscles are isolated for muscle regeneration analysis. (B) H&E staining of TA muscle for analysis of muscle fiber regeneration. The graph in the right panel shows the quantification of muscle fiber diameter in newly generated TA muscles, each observed in four different mice per group. (C) H&E staining of the liver for pathological analysis. (D) Blood biochemistry analysis. Con, Control group; CD, CD-treated group.

Encouraged by the results from in vitro study, the effect of CD on mouse muscle generation was investigated in vivo. First, mice were injured by BaCl_2_ injection on TA muscle, and CD or vehicle was administered (Fig. 5A). The histological analysis showed that the diameter of muscle fibers decreased significantly in CD-treated group (Fig. 5B). In addition, the lack of toxicity information regarding the co-administration of CD and BaCl_2_ prompted us to investigate its potential toxicity in the mouse liver and blood. The histopathologic analysis based on H&E staining (Fig. 5C) and blood biochemistry (Fig. 5D) did not show significant differences between CD and BaCl_2_ co-treatment group and the control group.

## Discussion

Aging is a process of physiological changes that occurs with age and refers to a decline in an organism's biological functions and ability to respond to stress. This process is accompanied by a decrease in skeletal muscle mass and strength (Degens and Alway, 2006; Kadi and Ponsot, 2010). The rapid loss of muscle mass due to aging is referred to as sarcopenia (Mithal et al., 2013; Rosenberg, 1997), and it is known to lead to exacerbate other diseases (Bielecka-Dabrowa et al., 2020; Di Monaco et al., 2022; He et al., 2021; Mesinovic and Scott, 2019; Mesinovic et al., 2019; Pan and Xu, 2022; Yeung et al., 2019).

Therapeutic agents derived from natural products have traditionally been used to treat human diseases and maintain health. Recent research has begun to provide scientific validation for the use of bioactive compounds sourced from natural products. It is crucial to harness the potential of available natural products to develop innovative drugs for the treatment of the diseases. Moreover, there is a need to investigate the physiological activity of various natural product extracts and conduct in-depth research on their efficacy, safety, and potential side effects. So, we studied the potential role of CD, which was previously used as an antimalarial drug along with hydroxychloroquine (Basrani et al., 2024). We demonstrated a significant inhibitory effect of CD on myogenic differentiation. It is notable that CD reduced myogenic differentiation while not inhibiting the growth in either proliferation or differentiation conditions. CD not only reduced myogenic differentiation in C2C12 cells but also decreased the expression of myogenic differentiation marker genes (Fig. 2).

One of the mechanisms for myogenesis involves the regulation of expression and phosphorylation of insulin receptor substrate-1 (IRS-1). Binding of insulin to the insulin receptor or insulin-like growth factor (IGF-1) receptor, it triggers the phosphorylation of IRS-1 (Sun et al., 1991), activating the Phosphatidylinositol 3-kinase (PI 3-kinase) /AKT pathway (Schiaffino and Mammucari, 2011). This activation is crucial for promoting the differentiation of precursor muscle cells and the formation of myotubes. IRS proteins are cytoplasmic proteins responsible for relaying signals from insulin and IGF-1 receptors to downstream mediators, IRS-1 and IRS-2 are highly expressed proteins within the IRS family and share significant homology. However, the phenotypes observed in knockout mice provide strong evidence that each IRS protein serves nonredundant functions (Araki et al., 1994; Sun et al., 1995). IRS-1 plays an important role in mediating the metabolic effects of insulin and IGF-1 in skeletal muscle (Miki et al., 2001). IRS-1 possesses several serine/threonine phosphorylation sites that play a role in the functional regulation of IRS-1. The phosphorylation of serine residues on IRS-1 appears to be involved in positive or negative regulation of insulin signaling (Copps et al., 2010; Weigert et al., 2008). We identified the inhibition of IRS-1/AKT signaling by CD as a critical mediator responsible for the inhibition of myogenic differentiation as shown in Fig. 3A. One of the downstream signaling kinases of AKT that regulates adult skeletal muscle mass, is reported to be a protein required for maintaining muscle structure and force production (Marabita et al., 2016). While several phosphorylation sites in IRS-1 have been identified in relation to insulin signaling, a clear definition of the specific phosphorylation sites that activate and inhibit insulin signaling remains elusive. Our result showed that IRS-1 Ser-318 undergoes phosphorylation during insulin activation, and this phosphorylation is inhibited by CD, subsequently suppressing downstream signaling. This result aligns with the observation that serine 318 residue of IRS-1 is phosphorylated early in the insulin signaling process in C2C12 cells, resulting in the activation of downstream signaling (Weigert et al., 2008). Interestingly, it was observed that the phosphorylation of GSK3 and insulin-induced S6K was maintained in the presence of CD (Fig. 3B), suggesting that CD may selectively inactivate AKT signaling required for muscle growth rather than muscle structure maintenance.

Additionally, we observed that CD diminishes myogenic differentiation in muscle satellite cells (Fig. 4A and 4B), which plays a crucial role in muscle regeneration. Consequently, this leads to the suppression of muscle regeneration following muscle damage (Fig. 4C).

In summary, our findings establish CD as a substance that impedes both myogenic differentiation and muscle regeneration. CD inhibited the expression of genes that induce muscle differentiation and decreased myogenesis and regeneration without affecting myoblast cell viability. Based on our results, we suggest that CD, used as an antimalarial drug, may have the potential to induce sarcopenia by inhibiting muscle differentiation and regeneration.

## Figures and Tables

**Fig. 6.**
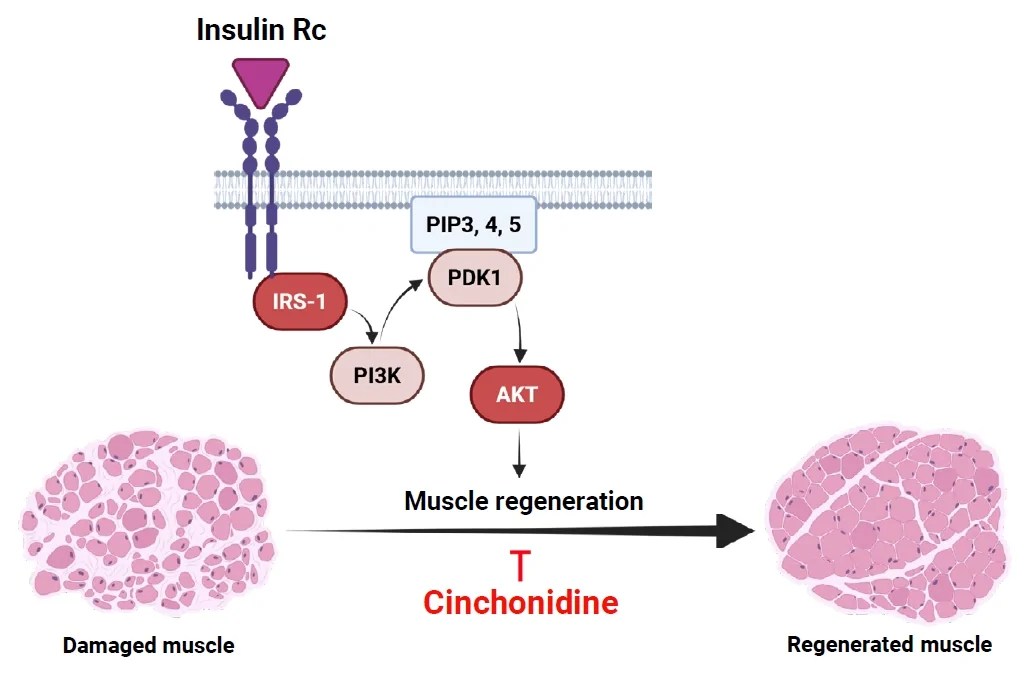
Schematic illustration depicting the mechanism of action of cinchonidine.

**Table 1.** PCR primers

Primer	Sequences
MCK-F	5'-CACCTCCACAGCACAGACAG-3'
MCK-R	5'-ACCTTGGCCATGTGATTGTT-3'
Myogenin-F	5'-CAACCAGGAGGAGCGAGACCTCCG-3'
Myogenin-R	5'-AGGCGCTGTGGGATATGCATTCACT-3'
GAPDH-F	5'-GCTTGTCATCAACGGGAAG-3'
GAPDH-R	5'-GATGTTAGTGGGGTCTCG-3'
